# The relationship between childhood psychological abuse and depression in college students: a moderated mediation model

**DOI:** 10.1186/s12888-024-05809-w

**Published:** 2024-05-30

**Authors:** Yang Liu, Qingxin Shen, Liangfan Duan, Lei Xu, Yongxiang Xiao, Tiancheng Zhang

**Affiliations:** 1 School of Sports Science, Jishou University, Jishou, China; 2 Institute of Physical Education, Shanxi University of Finance and Economics, Taiyuan, China

**Keywords:** Childhood psychological abuse, Depression, Alexithymia, Internet addiction, College students

## Abstract

**Background:**

Childhood psychological abuse (CPA) are highly correlated with depression among college students, but the underlying mechanisms between variables need further exploration. This study aims to investigate internet addiction as a mediating factor and alexithymia as a moderating factor, in order to further elucidate the potential risk factors between CPA and depression among college students.

**Methods:**

A self-report survey was conducted among 1196 college students from four universities in three provinces in China. The survey included measures of CPA, internet addiction, alexithymia, and depression. Descriptive and correlational analyses were performed on these variables, and a moderated mediation model was constructed.

**Results:**

CPA was positively correlated with depression among college students, as well as internet addiction with alexithymia. Internet addiction partially mediated the relationship between CPA and depression among college students, while alexithymia strengthened the relationships among the paths in the moderated mediation model.

**Conclusion:**

This study provides further insights into the psychological mechanisms underlying the relationship between CPA and depression among college students. Internet addiction serves as a mediating factor in this relationship, while alexithymia may enhance the strength of the relationships among the three variables.

## Introduction

Depression is a highly prevalent psychological disorder among young people, characterized by symptoms such as sadness, lack of energy, and despair [1]. Over the past decade, the incidence of depression has been continuously increasing [2, 3]. Studies show that the prevalence of depression among Chinese university students exceeds 25% [4, 5], and the global incidence rate is close to 30% [6]. Individuals with depression exhibit a variety of complex negative physical and mental manifestations [7]. Feelings of worthlessness, hopelessness, and self-blame are strong emotional experiences among depressed individuals [8]. Major cognitive impairments displayed by these individuals include emotional dysregulation, cognitive biases, difficulties in attention and memory, and inhibitory dysfunction [9, 10]. Similarly, outward behaviors manifest a range of negative patterns, such as social withdrawal [11], sleep disturbances [12], and abnormal changes in appetite [13]. These physical and mental manifestations further deepen the severity of depression [14]. Additionally, the etiology of depression is complex, with early-life stress being a significant risk factor [15], and that is often associated with adverse childhood experiences [16], such as childhood abuse. Childhood abuse has a close relationship with depression [17–20], and studies have found that among the subtypes of childhood abuse that childhood psychological abuse (CPA) is most closely related to depression [18, 21]. Given the significant harm of depression, social attention, and its strong association with CPA, this study strongly needs to explore the underlying mechanisms between the two, in order to intervene and prevent timely and predict the impact of CPA on individual depression.

CPA refers to inappropriate psychological parenting behavior that guardians continuously and repeatedly adopt during childhood, which has an adverse effect on individuals' growth [22]. Due to its particular nature, the detection rate of CPA is quite high in different countries [23, 24]. Abuse and neglect are two subtypes of CPA that are increasingly accepted by scholars in related studies. Longitudinal studies have found that parents' psychological neglect predicts future depression in adolescents [25]. In addition, research has found that among the classifications of childhood abuse, the correlation between psychological abuse and depression is the highest [18]. Based on the above review, this study hypothesized that CPA can significantly predict the occurrence of depression in college students.

Individuals who have experienced CPA often encounter emotional distress. Faced with such distress, they may be at an increased risk of engaging in hazardous behaviors, such as internet addiction, as these online activities may serve as a coping mechanism to alleviate negative emotions [26]. Without intervention, this reliance on the internet can form a cyclical pattern, potentially leading to internet addiction. Internet addiction is characterized by an excessive, problematic, and compulsive engagement in behaviors related to internet use [27, 28]. Research indicates that among the various subtypes of childhood maltreatment, CPA has the strongest association with internet addiction [29]. CPA can significantly predict individuals internet addiction, and internet addiction has been found to mediate the relationship between CPA and suicidal internet addiction behaviors [30]. In discussions that integrate the relationship between childhood maltreatment and internet addiction, CPA is highlighted as a particularly salient predictor [31]. Consistent with the social compensation theory [32], CPA may lead individuals to seek emotional fulfillment through online interactions. Furthermore, there is a recognized association between internet addiction and depression. Research has found a strong correlation between internet addiction and depression [33], with internet addiction being a significant predictor of depression [34]. Longitudinal studies have shown a significant bidirectional relationship between internet addiction and depression among college students [35]. The displacement hypothesis [36] suggests that excessive internet use may impede real-life social interactions, reduce well-being, and deepen depression. Depression can also intensify the level of internet addiction, creating a vicious cycle and leading to a "rich get richer" scenario [37]. Based on this evidence, this study posits that CPA can significantly predict internet addiction among college students, which in turn can significantly predict depression.

However, when individuals possess certain traits, the relationships among the variables mentioned above may be strengthened, exacerbating negative behaviors or psychological outcomes. Among these variables, the level of alexithymia is one of the more important ones. Alexithymia is a stable personality trait [38], characterized by limited ability to understand one's own feelings and others' emotions, inadequate emotion regulation in interpersonal interactions [39], difficulty in recognizing emotions, describing emotions, and an externally oriented thinking style [40]. Alexithymia, due to emotional dysregulation, can lead to the intensification of negative emotions such as anxiety and depression [41], and inaccurate attention and expression of emotions may result in poor interpersonal relationships [42], thereby increasing individuals' psychological burden. To escape or alleviate such negative psychological states, the internet on mobile phones provides an easily accessible avenue [42–45]. According to the alexithymia stress hypothesis, individuals with high levels of alexithymia often find themselves in a state of stress due to their inadequate understanding and recognition of their own and others' emotions [46], which further predicts severe negative psychological states [47]. Therefore, based on the aforementioned review, it is evident that alexithymia may enhance the relationship between CPA, internet addiction, and depression discussed in this study, further exacerbating the degree of negative psychological and behavioral outcomes. Additionally, individuals with alexithymic characteristics not only neglect emotions [48], but they may also have a generalized impairment in perceiving internal bodily sensations (interoception) compared to individuals with lower levels of alexithymia [49], as demonstrated by various studies on the accuracy of perceiving heart rate [50, 51], delayed healthcare seeking for illnesses [52], and unstable substance intake [53], among others. Based on these features, individuals with high levels of alexithymia tend to overlook their own discomfort symptoms even when they excessively use the internet [54, 55] due to their lower sensory perception. Therefore, we hypothesize that alexithymia moderates the relationships among CPA, internet addiction, and depression mediated by various paths.

**Fig. 1:**
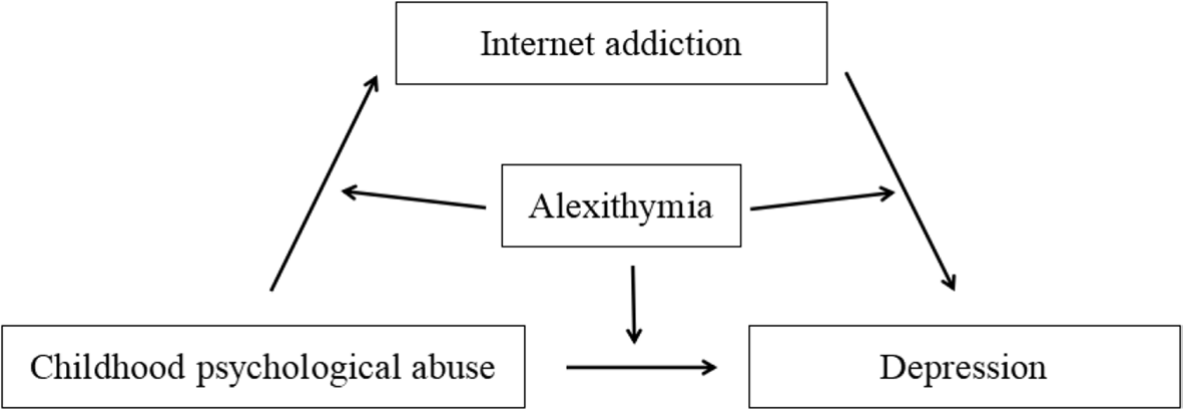
Hypothesized a moderated and mediation model

In summary, previous research strongly indicates the relationship and predictive role of CPA and depression, but these areas are relatively understudied among Chinese university students. To further supplement research in this field and explore underlying psychological mechanisms, this study introduces the mediating variable of internet addiction and the moderating variable of alexithymia. Therefore, this study constructs a hypothetical path model (see Fig. 1).

## Methods

### Participants

This cross-sectional survey was conducted in October 2023 among Chinese university students from four universities in Hunan Province, Hubei Province, and Guangxi Province. Prior to distribution, the researchers delivered a presentation to all participants, informing them of the main content and confidentiality of the survey data, as well as its ultimate purpose. The electronic questionnaires were distributed on a class basis, with an informed consent statement attached to the questionnaire's cover page. Participants could proceed with the survey only after choosing to agree, while those who declined would be directed to an exit page. Informed consent was obtained from all the participants. The survey was anonymous and voluntary, and it could be completed within 20 min. Prior to commencement, this study obtained approval from the Biomedicine Ethics Committee of Jishou University. We confirm that all the procedure is in accordance with the relevant guidelines and regulations such as the declaration of Helsinki. A total of 1352 students completed the survey, and after excluding respondents with excessively short response times or patterns in their answers, valid data from 1196 participants (496 males, 700 females) were ultimately obtained, with an average age of 18.69 years (SD = 1.07).

## Measures

### Childhood psychological abuse (CPA)

The measurement of CPA utilized the psychological abuse and neglect subscales from the Short Childhood Trauma Questionnaire (SCTQ) [56]. Each subscale included 5 items, scored on a Likert scale of 1 (never) to 5 (always), assessing the experiences of participants before the age of 17. An example item from the scale is: “Someone in my family said insulting or sad things to me”. Higher scores indicated higher levels of CPA. In this study, the Cronbach's α for the sample was 0.878.

### Depression

Depression among college students was measured using the depression subscale from the Chinese version of the Depression Anxiety Stress Scale (DASS-21) [57]. The subscale comprised 7 items, scored on a Likert scale of 1 (strongly disagree) to 4 (strongly agree), assessing the level of depression experienced by participants. An example item from the scale is: “I can't be enthusiastic about anything”. Higher scores indicated more severe depression. In this study, the Cronbach's α for the sample was 0.906.

### Internet addiction

Internet addiction among college students was measured using the Problematic Social Media Use (PSMU) Scale [58]. The scale comprised 8 items, scored on a Likert scale of 1 (not at all) to 5 (completely), assessing the level of internet addiction experienced by participants. An example item from the scale is: “Using social networking sites distracts me from my studies”. Higher scores indicated more severe internet addiction. In this study, the Cronbach's α for the sample was 0.857.

### Alexithymia

The Toronto Alexithymia Scale (TAS-20) was used to assess the level of alexithymia among college students [59]. The scale comprised 20 items, scored on a Likert scale of 1 (totally disagree) to 5 (totally agree), assessing the level of alexithymia experienced by participants. An example item from the scale is: “I am often confused about what emotion I am feeling”. Higher scores indicated more severe alexithymia. In this study, the Cronbach's α for the sample was 0.804.

### Covariates

Considering the potential influence of demographic variables, such as gender and age [31, 60], on the analysis results, we controlled for these variables in our analysis.

### Statistical analyses

All statistical analyses were conducted using SPSS 26.0 software. Firstly, we checked for methodological biases to evaluate the potential bias resulting from self-report questionnaires. Before initiating the data analysis, we assessed the normality of our data using the Shapiro–Wilk test. According to Kim's proposal, data exhibiting an absolute skewness value below 2 and an absolute kurtosis value below 7 may be deemed to approximate a normal distribution [61]. In our study, we found that the variables CPA, depression, internet addiction, and alexithymia were normally distributed. For variables conforming to a normal distribution, descriptive analysis was conducted using the mean and standard deviation (Sd), while Pearson's correlation analysis was employed to assess the relationships among them. Then, we standardized the data of the main variables before conducting the analyses. Finally, to test our hypotheses, we used the PROCESS macro (Mode 4 and Model 59) in SPSS to analyze the relationships between variables [62]. The PROCESS macro was based on a bootstrapping method with 5000 resamples to estimate the model testing and 95% confidence intervals (95% CI), and a relationship was considered significant when the 95% CI did not include 0. Gender and age were considered as covariates in the analyses, and the significance level was set at α = 0.05.

## Results

### Harman’s single factor test and normality test

Harman's single-factor test was used to examine the impact of common method bias. The analysis results showed that there were 2 factors with eigenvalues greater than 1. Without rotating the principal component factors, the explanatory rate of the first factor was 35.55%, which is lower than the recommended threshold of 40% [63]. Therefore, this study did not encounter severe common method bias. Upon assessing normality for our principal variables, all variables exhibited absolute skewness values below 2 and absolute kurtosis values below 7. Consequently, parametric tests were employed for all subsequent analyses.

### Descriptive analyses

The results of Table 1 show that CPA (t = 2.62, *p* < 0.001), depression (t = 2.32, *p* < 0.05) and Internet addiction (t = -2.17, *p* < 0.05) are different between genders and reach statistical significance.

**Table 1:** Descriptive results

Variables	Total (*n* = 1196)		Male (*n* = 496)		Female (*n* = 700)		Male vs. Female
	Mean	Sd	Mean	Sd	Mean	Sd	t
CPA	20.03	7.01	20.66	6.97	19.57	7.16	2.62***
Depression	12.48	4.33	12.83	4.62	13.23	4.09	2.32*
Internet addiction	22.14	6.29	21.67	6.72	22.47	5.96	-2.17*
Alexithymia	53.02	9.75	53.08	9.88	52.98	9.66	0.18

*CPA* Childhood psychological abuse

^*^p < 0.05

^***^p < 0.001

### Correlational analyses

Table 2 presents the Pearson correlation data between the variables of interest. CPA was significantly positively correlated with college students' internet addiction (*r* = 0.240, *p* < 0.001), depression (*r* = 0.481, *p* < 0.001), and alexithymia (*r* = 0.322, *p* < 0.001). College students' internet addiction was significantly positively correlated with depression (*r* = 0.384, *p* < 0.001) and alexithymia (*r* = 0.262, *p* < 0.001). Depression was significantly negatively correlated with college students' alexithymia (*r* = 0.461, *p* < 0.001).

**Table 2:** Correlational analyses (*n* = 1196)

	Age	CPA	Internet addiction	Depression	Alexithymia
Age	-				
CPA	0.188***	-			
Internet addiction	0.035	0.240***	-		
Depression	0.129***	0.481***	0.384***	-	
Alexithymia	0.018	0.322***	0.262***	0.461***	-

^***^p < 0.001

### Mediation analysis

Table 3 presents the results showing that, after controlling for gender and age, CPA can significantly predict depression in college students (β = 0.473, SE = 0.026, *p* < 0.001). When internet addiction was included as a mediator variable, CPA continued to significantly predict depression in college students (β = 0.402, SE = 0.025, *p* < 0.001). Additionally, upon testing the mediation model, it was found that CPA significantly predicts internet addiction in college students (β = 0.245, SE = 0.029, *p* < 0.001), and internet addiction also significantly predicts depression (β = 0.290, SE = 0.025, *p* < 0.001).

**Table 3:** Mediation model test

Variables	Depression			Internet addiction			Depression		
	β	SE	t	β	SE	t	β	SE	t
Gender	-0.049	0.053	-0.921	0.170	0.059	2.889**	-0.098	0.051	-1.940
Age	0.032	0.025	1.269	0.009	0.028	0.337	0.029	0.024	1.225
CPA	0.473	0.026	18.300***	0.245	0.029	8.576***	0.402	0.025	15.927***
Internet addiction							0.290	0.025	11.660***
R^2^	0.233			0.064			0.312		
F	120.941***			27.351***			134.967***		

^**^p < 0.01

^***^p < 0.001

### Moderated and mediation analysis

**Fig. 2:**
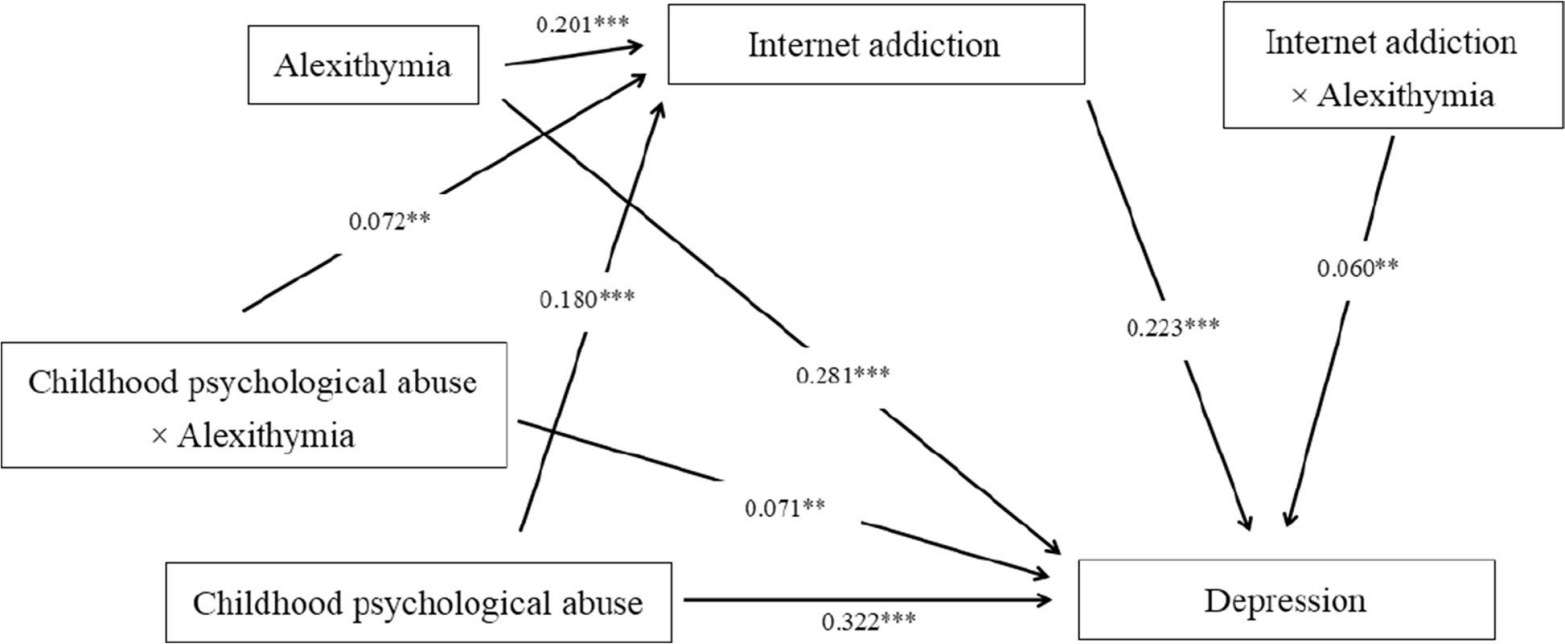
Moderated and mediation model

**Fig. 3:**
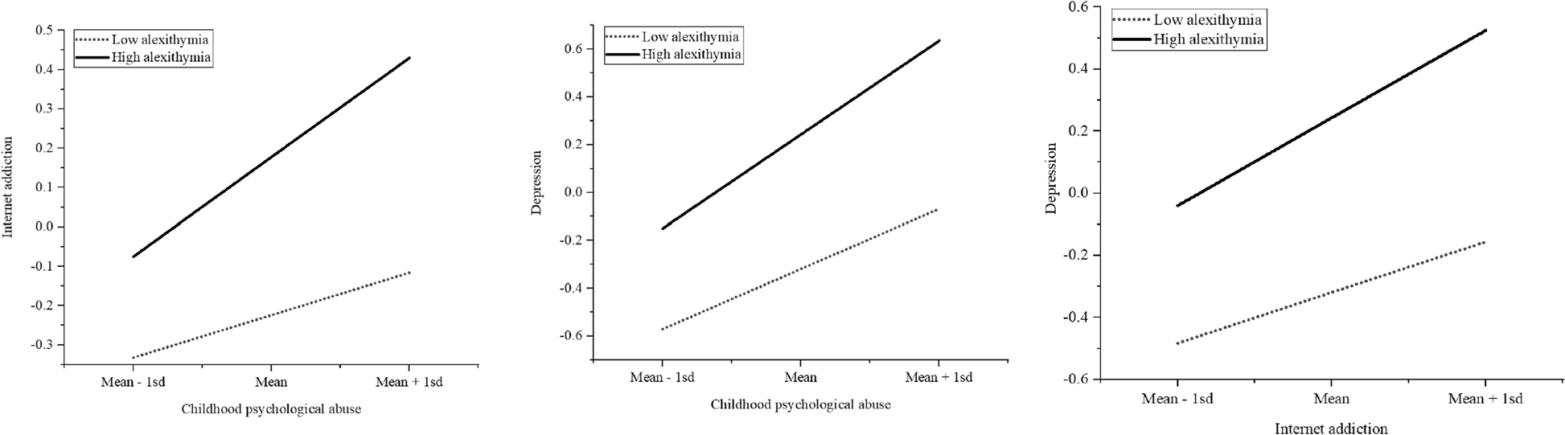
Simple slope plot

After controlling for covariates, the moderated mediation model analysis revealed that the predictive effects of all paths in the mediation model remained significantly present (CPA predicting depression:β = 0.322, SE = 0.025, *p* < 0.001; CPA predicting internet addiction:β = 0.180, SE = 0.030, *p* < 0.001; Internet addiction predicting depression:β = 0.223, SE = 0.024, *p* < 0.001). Additionally, alexithymia significantly predicted college students' internet addiction (β = 0.201, SE = 0.029, *p* < 0.001) and depression (β = 0.281, SE = 0.025, *p* < 0.001). Lastly, the interaction term between alexithymia and CPA significantly predicted college students' internet addiction (β = 0.072, SE = 0.027, *p* < 0.01) and depression (β = 0.071, SE = 0.023, *p* < 0.01), and the interaction term between internet addiction and alexithymia significantly predicted college students' depression (β = 0.060, SE = 0.022, *p* < 0.01). Refer to Table 4, Figs. 2 and 3 for details.

**Table 4:** Moderated and mediation analysis

	Internet addiction			Depression		
	β	SE	t	β	SE	t
Gender	0.157	0.058	2.725**	-0.101	0.047	-2.140*
Age	0.018	0.027	0.654	0.040	0.022	1.817
CPA (A)	0.180	0.030	6.100***	0.322	0.025	13.087***
Alexithymia (B)	0.201	0.029	6.921***	0.281	0.025	11.390***
A X B	0.072	0.027	2.728**	0.071	0.023	3.051**
Internet addiction (C)				0.223	0.024	9.370***
B X C				0.060	0.022	2.707**
R^2^	0.108			0.400		
F	28.736***			113.278***		

^*^p < 0.05

^**^p < 0.01

^***^p < 0.001

## Discussion

This study examines the relationships between CPA, internet addiction, depression, and alexithymia among college students. The findings reveal positive correlations between CPA, internet addiction, depression, and alexithymia, all of which are statistically significant. After controlling for demographic variables, internet addiction is found to mediate the relationship between CPA and depression in college students, while alexithymia moderates this relationship, confirming our initial hypothesis.

Our study confirms the positive correlation between CPA and depression in college students, which is consistent with previous research [18, 31]. Studies conducted in China have shown that childhood abuse is relatively common [64], with emotional abuse being the most prevalent type [65, 66]. Almost all types of childhood abuse are associated with mental health problems [20, 23, 67, 68], increasing the risk of various adversities in individuals' later lives [23, 69, 70], with psychological abuse being particularly prominent [31, 68], even predicting somatic symptoms in patients with severe depression [71]. Furthermore, research has found that CPA becomes the sole predictor of adolescent depression when controlling for other types of abuse, and early psychological abuse has a greater impact on depression [72]. Children who receive warm, rule-following, and well-bounded care from parents perform better in various aspects, including mental health [73]. On the other hand, children who experience psychological abuse from caregivers, in a state of invisible stress similar to social isolation, are more likely to develop depression, anxiety, and even aggressive behaviors [74].

Our study supports the hypothesis that internet addiction mediates the relationship between CPA and depression in college students, which is consistent with other similar studies [29]. Previous research has found a strong association between CPA and internet addiction among young people [30, 31, 75, 76]. The relationship between internet addiction and depression has also been strongly supported [77], including in studies conducted in China [35]. According to the explanations of social control theory [78] and compensatory internet use theory [32], CPA seems to indicate unfavorable family support environments for adolescents, leading them to seek support from the virtual online world and developing internet addiction. It was found that adolescents who were addicted to the internet at baseline were more likely to develop depression in the future [77], which is also true in other studies [79]. Adolescents addicted to the internet often face greater stress, making them more prone to depression [80].

As previously hypothesized, alexithymia strengthens the relationships between all pathways. Individuals with alexithymia have difficulty understanding their own and others' emotions and are unable to regulate their emotions appropriately in daily interpersonal interactions [39], leading to an exacerbation of negative emotions [41]. Additionally, based on the explanations of general strain theory [81] and compensatory internet use theory [82], individuals with alexithymia feel stressed in dealing with interpersonal relationships [83]. Under this negative emotional state, in order to meet the needs of interpersonal communication and escape from the pressure of reality, they naturally choose the online world, which further leads to the development and intensification of internet addiction. However, alexithymia's "dullness" towards one's own feelings is not limited to emotions but can also extend to the perception of internal sensations [49]. Brain regions associated with these internal sensations include the anterior insula and anterior cingulate cortex [84, 85], which not only play a role in non-emotional interoception but also have significant implications for individuals' emotional processing [86, 87]. Studies have found structural abnormalities in these brain regions in individuals with alexithymia [88–90]. It is possible that individuals with severe pain and fatigue continue to use the internet despite their condition, further exacerbating their involvement in negative psychological and behavioral patterns. Therefore, high levels of alexithymia strengthen the relationships between CPA, internet addiction, and depression, which aligns with the expectations of this study.

In conclusion, our study further contributes to understanding the relationship between CPA and depression in college students, as well as the mediating role of internet addiction and the moderating role of alexithymia. These findings are not commonly seen in previous research. However, the study has several limitations. Firstly, the accuracy of self-reported CPA data may be insufficient as it involves retrospective self-reporting. Secondly, the representativeness of the sample may be inadequate as we only selected local colleges from a few provinces in China, with most of the students being locals. Future studies could increase the diversity of the sample. Lastly, due to the cross-sectional nature of the study, the causal relationships between variables are challenged. Therefore, future research could explore causal relationships based on longitudinal tracking.

## Conclusion

This study discusses the relationships between CPA, internet addiction, depression, and alexithymia among college students, confirming the mediating role of internet addiction and the moderating role of alexithymia between CPA and depression. Individuals, families, schools, and society should pay attention to the negative impacts caused by CPA, especially for individuals with high levels of alexithymia.

## Data Availability

The datasets generated and/or analysed during the current study are not publicly available due [our experimental team's policy] but are available from the corresponding author on reasonable request.
